# Can illustrating updated information help prevent the activation of outdated information?

**DOI:** 10.1007/s00426-026-02417-z

**Published:** 2026-11-04

**Authors:** Anne Schüler, Ekaterina Blinova, Pauline Frick

**Affiliations:** 1 Leibniz-Institut für Wissensmedien, Schleichstraße 6, 72076 Tübingen, Germany; 2 Humboldt-Universität zu Berlin, Berlin School of Mind and Brain, Unter denLinden 6, 10099 Berlin, Germany; 3 Eberhard Karls Universität Tübingen, Tübingen, Germany; 4 Tübingen Center for Digital Education, Wilhelmstr. 31, 72074 Tübingen, Germany

## Abstract

Research has shown that outdated information can influence reading processes, even if it is no longer true or relevant in the current context. In two online experiments, we investigated whether illustrating the updated information reduces the activation of outdated information. Participants read short texts in which initial information was later updated. We varied within-participants whether the updated information was accompanied by an illustration or not. After a background section, participants read a target sentence referring to the updated information. We measured response times to verification statements related to the outdated information, presented either before or after the target sentence. If outdated information is activated during reading the target sentence, participants should respond faster to related statements after the target sentence than before. If illustrating the updated information reduces the activation of the outdated information, this effect should diminish with the presentation of illustrations. However, contrary to our expectations, no such effect was observed: the illustrations had no impact on the activation of outdated information, neither in Experiment 1 (*N* = 322), where the updated information was presented in a straightforward manner, nor in Experiment 2 (*N* = 357), where it was additionally supported by causal explanations. Instead, in both experiments, we found that participants responded faster to the verification statements after the target sentence than before it, regardless of whether the updated information was illustrated. Hence, it appears that illustrating the updated information is not an effective way to prevent the activation of outdated information.

## Public significance statement

Misinformation is a major societal challenge because it can continue to influence how people think and understand new information, even after it has been corrected.

In our study, adding illustrations to updated information did not prevent outdated or false information from being mentally activated during reading.

These findings highlight how difficult it is to counter misinformation effectively and suggest that stronger or additional strategies are needed to ensure that corrections truly replace false beliefs.

## Introduction

One of the central challenges of our time lies in dealing with misinformation, which is particularly widespread on social media platforms and blogs (e.g., Aïmeur et al., 2023; Clayton et al., 2020; Lewandowsky et al., 2012). Ecker et al. (2022, p.13) define misinformation “as any information that turns out to be false”. A key concern is that such misinformation can remain mentally accessible and influence the processing of new information—even after it has been explicitly refuted (Ecker et al., 2022). One approach to investigating the cognitive processes underlying this persistent influence is to study the updating of information during reading. In this line of research, information that is initially valid within a text but is rendered inapplicable by subsequently presented updates is referred to as *outdated information*. Although outdated information is not necessarily misinformation, both require recipients to revise previously encoded mental representations to incorporate updated information, and in both cases, the initial information may remain accessible and continue to influence subsequent processing. Thus, studying how readers update outdated information may provide insights into basic cognitive processes that are also relevant to reducing the influence of misinformation (cf. Kendeou, 2024).

Based on previous research suggesting that enriching updated information may reduce the influence of outdated information, the present research focuses on whether illustrations can serve as an effective means of supporting this process. This research question is inspired by reading research, where Kendeou et al. (2013) demonstrated across a series of seven experiments that supplementing updated information with causal explanations can significantly reduce or even completely eliminate the impact of outdated information. A plausible explanation for this finding is that causal information tends to support the construction of a rich and elaborated knowledge network (cf. Brownstein & Read, 2007; Trabasso & Suh, 1993). The combination of updated information with causal explanations may thus foster the development of an elaborated mental network that is associated with a high level of activation. This, in turn, reduces the activation level of the outdated information, thereby weakening its influence (Kendeou, 2024).

Building on this research, the present paper examines whether illustrations that visually represent the updated information may provide another means of supporting updating. Research in the field of learning with illustrated texts (Mayer, 2021) suggests that combining text with illustrations leads to a more elaborated mental model than text alone. This leads to the hypothesis that illustrations—similar to causal explanations—may help build a mental network that generates stronger activation and thus reduces or eliminates the activation of outdated information. Clarifying whether illustrations can support these processes is also of practical relevance, given that illustrations have been recommended as a means of correcting misconceptions (e.g., Brashier & Marsh, 2020). Although previous research has examined the role of illustrations in related contexts, such as refutation text (Danielson et al., 2016; Mason et al., 2017) and debunking misinformation (Dixon et al., 2015; Newman et al., 2012; Whitehead et al., 2025), to our knowledge, only one study has specifically investigated whether illustrations reduce the activation of outdated information during reading, as reflected in reading times (Frick et al., 2025). Their results indicated that illustrations facilitated text processing but did not specifically reduce interference from outdated information. Given the absence of the expected effect in previous research, the overarching aim of the present experiments was to further examine whether illustrations can reduce the activation of outdated information during reading. More specifically, our aims were twofold: First, we sought to test the original hypotheses by Frick et al. (2025) using a different paradigm (Experiment 1). Second, we aimed to examine whether combining illustrations with causal explanations – the latter previously identified as effective by Kendeou et al. (2013) – constitutes an effective strategy for suppressing the activation of outdated information (Experiment 2).

### Research on updating text information

The term *updating* refers to the modification of an existing mental representation in response to newly encountered information during text comprehension. The process of updating can be illustrated by the following example (see also O’Brien & Cook, 2016b): A text initially describes how Terry (the protagonist) decides to purchase an oboe. However, as the text progresses, she revises her decision and buys a cello instead. Readers initially integrate Terry’s intention to purchase an oboe into their mental representation of the described situation. When they subsequently encounter the updated information that Terry has decided to buy a cello, this new information reactivates the related information about the oboe. The two pieces of information are then integrated and validated with respect to their compatibility, prompting an update of the mental representation to reflect Terry’s revised decision. Consequently, the initial information about the oboe becomes outdated information, whereas the information about the cello represents the updated information. However, both pieces of information may remain represented in memory, and their relative activation determines which information is more accessible and therefore more likely to guide subsequent comprehension and retrieval. These processes are described in greater detail below in the context of the Resonance–Integration–Validation (RI-Val) Model of Comprehension (O’Brien and Cook 2016a) and the Knowledge Revision Components Framework (KReC; Kendeou, 2024; Kendeou & O’Brien, 2014).

As the example illustrates, the process of updating concerns newly introduced text information and can therefore be examined independently of recipients’ topic-related prior knowledge. However, it is assumed that the underlying cognitive mechanisms are similar to those involved in *knowledge revision* or *conceptual change*, where recipients already possess topic-related prior knowledge (e.g. Sinatra, & Broughton, 2011; Vosniadou, 1994; cf. Kendeou, 2024). Therefore, processes observed during updating are also considered relevant for knowledge revision or conceptual change. For updated information to guide subsequent comprehension effectively, its activation should outweigh that of the outdated information. Empirical evidence shows, however, that outdated information frequently remains influential: even when outdated information has been explicitly refuted, it often becomes automatically activated (i.e., without the reader’s active involvement) when subsequent text content shows thematic or even only superficial overlap with the outdated content (e.g., Guéraud et al., 2005; Kendeou et al., 2013; O’Brien et al., 1998, 2010).

The continued activation of outdated information is reflected in longer reading times for target sentences referring to updated information. For example, readers are slower to process “Terry showed her friend the cello she bought” when Terry initially intended to buy an oboe than when she intended to buy a cello from the outset (Guéraud et al., 2018; O’Brien et al., 1998, 2010). This disruption is attributed to the concurrent activation of updated and outdated information (Kendeou et al., 2013).

Importantly, as Frick et al. (2025) argue, longer reading times in the outdated condition do not necessarily imply that readers have failed to update their mental representation (cf., O’Brien et al., 2010). In fact, many participants are able to correctly report that Terry bought a cello. Thus, successful updating and the continued activation of outdated information are not mutually exclusive. Rather, longer reading times indicate that outdated information can continue to influence text comprehension and inferential processing—even when readers have already adjusted their mental models accordingly (Ecker et al., 2015; Lewandowsky et al., 2012; Rich & Zaragoza, 2016).

Although our primary focus is on research examining updating during text comprehension, it is worth noting that the persistence of outdated information observed in these paradigms parallels findings from research on the continued influence effect. The continued influence effect describes the finding that misinformation can continue to shape reasoning, judgment, or decisions even after it has been corrected and accepted as true (Ecker et al., 2022; Lewandowsky et al., 2012; Walter & Tukachinsky, 2020). It has been demonstrated using misinformation about both fictitious events, such as the causes of fires or accidents (e.g., Wilkes & Leatherbarrow, 1988), and real-world issues, such as vaccination (Pluviano et al., 2022).

An early demonstration of this effect was provided by Wilkes and Leatherbarrow (1988). Participants read a report in which a warehouse fire was initially attributed to gas cylinders and oil paints stored in a side room. Some participants later received a retraction stating that the side room had been empty before the fire. Nevertheless, their subsequent explanations continued to refer to the retracted materials as a possible cause. Thus, as in the research examining updating during text comprehension, the later correction was encoded without rendering the original information mentally unavailable or preventing it from influencing subsequent reasoning.

The two research traditions share a common underlying structure: Initially presented information is incorporated into a mental representation, subsequently contradicted or corrected, and later becomes relevant again. In both cases, the original information may remain accessible despite successful encoding of the updated information. However, the research traditions differ in their typical emphasis. Updating research often examines the online reactivation of outdated information during subsequent text processing, for example through reading-time measures, whereas research on the continued influence effect typically assesses whether explicitly corrected misinformation continues to influence subsequent explanations, inferences, beliefs, or judgments. As discussed in greater detail below, both lines of research also examine how the continued influence of outdated information or misinformation can be reduced.

### Theoretical accounts of updating during text comprehension

Theoretically, the process of updating during text comprehension can be explained by automatic activation processes, as described in numerous memory-based theories (Albrecht & O’Brien, 1993; Kintsch, 1998; Myers & O’Brien, 1998). Although these theories were developed primarily to explain language and text comprehension, their assumptions may also inform our understanding of why corrected misinformation remains influential and how its influence can be reduced.

Within the framework of the RI-Val model (O’Brien and Cook 2016a), activation represents one of three automatic processes that occur during reading (i.e., activation, integration, and validation). According to the model, activation operates via a passive resonance mechanism: the information currently being read and entering working memory functions as a set of cues that probe long-term memory. Information from the previous discourse context (e.g., Albrecht & O’Brien, 1993) or from prior knowledge (Beker et al., 2017) that shares features with these cues is activated through this process (O’Brien et al., 1998). Cook and O’Brien (2014) describe this activation mechanism as “dumb,” since any information that even partially overlaps with the cues can be activated. This means that irrelevant, incorrect, or outdated information can also be automatically activated, as long as it bears some similarity to the information currently being processed. The most strongly activated information then enters working memory and is integrated with the newly acquired information. If both updated and outdated information are activated, they will be integrated with each other and with the current information already present in working memory. In a subsequent validation process, the system evaluates whether the resulting connections are coherent. Validation is assumed to operate routinely in the background as an integral part of comprehension (Richter, 2015). Empirical studies suggest that it is a routine, nonstrategic process (Richter et al., 2009): Readers appear unable to ignore the validity of information even when validity is irrelevant or even detrimental to the experimental task (Isberner & Richter, 2014). Validation processes may also operate across modalities, for example, by evaluating the consistency between verbal and pictorial information (Schüler & Frick, 2024). In sum, the processes specified within the RI-Val model help explain why outdated information may still be activated even after it has been discredited by updated content in the text and why outdated information may lead to disruptions in the reading process and hinder comprehension (Johnson & Seifert, 1994; Lewandowsky et al., 2012; O’Brien et al., 1998, 2010; Rich & Zaragoza, 2016). These assumptions may also help explain the continued influence of misinformation: Previously encountered misinformation may be reactivated when subsequent information overlaps with it, even after a correction has been encoded (Lewandowsky et al., 2012; Rich & Zaragoza, 2016). Thus, theories of language and text processing provide a process-level account of why corrected misinformation may remain accessible and continue to affect subsequent comprehension and reasoning.

Another theoretical model, the KreC framework (Kendeou, 2024; Kendeou & O’Brien, 2014), builds upon these assumptions but goes a step further by describing additional conditions that are necessary for knowledge revision during reading. Although the framework focuses on knowledge revision, its assumptions also have implications for the persistence of outdated information. In line with the RI-Val model, the KReC framework assumes that once knowledge has been encoded, it cannot be completely erased from long-term memory and may be automatically activated when related information is presented. Thus, outdated information may remain accessible and influence processing even after updated information has been encountered. To enable successful knowledge revision, three conditions are key: co-activation, integration, and competing activation. Initially, both outdated and updated information must be simultaneously activated and then integrated with one another. This means that the two pieces of information are linked in memory or embedded within a mental network. According to the principle of competing activation, knowledge revision is facilitated to the extent that cognitive activation is redirected from outdated to updated information. The more updated information is available, and the more interconnected this information is, the more effectively activation shifts away from the outdated content. As a result, the activation of outdated information is reduced, diminishing its disruptive influence on text processing. Applied to the correction of misinformation, these assumptions suggest that a correction should not merely present accurate information but should establish a sufficiently strong and interconnected representation that can compete with the misinformation during subsequent processing. Accordingly, enriching corrective information may reduce the influence of misinformation by increasing the relative activation and accessibility of the updated representation.

### Influence of textual features on updating information

Empirical studies have primarily focused on how textual features influence competing activation as proposed by the KReC framework and, consequently, knowledge revision (Ecker et al., 2015; Guéraud et al., 2005, 2018; Johnson & Seifert, 1994; Kendeou et al., 2013, 2014, 2019; for exceptions, see the next chapter). Of particular relevance to the present experiments, Kendeou et al. (2013) examined whether causal explanations reduce the activation of outdated information using the same type of statement-verification paradigm employed in the present study. In their experiments, the participants read texts with outdated information. The presence or absence of a causal explanation for the update was manipulated. The results showed that the addition of just a single causal sentence was sufficient to eliminate any disruption in target sentence reading time caused by outdated information. However, the absence of prolonged reading times does not necessarily indicate that the outdated information is no longer activated. To examine this, Kendeou et al. used response times in a statement verification task instead of target sentence reading times as the dependent variable (for a similar procedure, see Guéraud et al., 2005, Experiment 3). For this purpose, verification statements referring to the outdated information were presented either immediately before or after the target sentence referring to the updated information. If the outdated information is automatically activated when reading the target sentence, participants should respond more quickly to the verification statement presented after rather than before the target sentence because the relevant information is already active in memory. Conversely, if the outdated information is not activated during target sentence processing, no such difference in response times should occur. According to Kendeou et al. (2013), this paradigm should be more sensitive to the activation of the information than target sentence reading times. The results showed that the outdated information was still activated even when one causal sentence was added, as participants responded more quickly to verification statements presented after rather than before the target sentence. In subsequent experiments, Kendeou et al. (2013) increased the amount of causal information from one causal sentence to three. With three causal sentences, no measurable activation of the outdated information was observed in the statement verification task as response times no longer differed depending on whether the verification statement was presented before or after the target sentence (see also Kendeou, 2024). The positive effect of causal explanations may be attributed to the fact that they provide a rich elaborative network. This elaboration strengthens the activation of the updated information while simultaneously suppressing the activation of outdated information.

The findings of Kendeou et al. (2013) can be situated within the broader literature on refutation texts. Refutation texts are specifically designed to address misconceptions. Refutation texts follow a specific structure: They first activate the relevant misconception, then explicitly refute it, and subsequently present the correct explanation (Mason et al., 2019; Tippett, 2010). The effectiveness of refutation texts can be explained within the KReC framework. By explicitly presenting both the misconception and the correct information, refutation texts facilitate their coactivation and integration. The subsequent explanation embeds the correct information within a coherent network of related information, thereby strengthening its activation and supporting a shift in competing activation away from the misconception and toward the correct information. Consequently, the misconception may remain represented in memory but should be less likely to interfere with subsequent comprehension. Meta-analyses confirm the positive effects of refutation texts in addressing misconceptions (Danielson et al., 2025; Schroeder & Kucera, 2022).

In the context of debunking misinformation (i.e., reactive interventions after misinformation has been introduced; cf. Ecker et al., 2022), empirical evidence also suggests that corrections can more effectively reduce the continued influence of misinformation when they provide a plausible alternative explanation rather than merely negating the misinformation (Ecker et al., 2010; Johnson & Seifert, 1994; Swire et al., 2017).

Taken together, research on text updating, refutation texts, and misinformation debunking indicates that textual features such as causal explanations can support updating by strengthening updated or correct information and reducing the influence of outdated or incorrect information. Beyond these textual features, other factors may also influence whether outdated information is activated (Ecker et al., 2022), including the presence of accompanying illustrations.

### Influence of illustrations on updating information

As outlined above, it is assumed that the elaboration of the updated information can reduce the activation of outdated information. In addition to textual features, illustrations that illustrate updated content may also contribute to building such a rich elaborative network. First, illustrated texts allow for dual coding of information—that is, encoding both in verbal and pictorial formats (see, e.g., Paivio, 1990). Second, it is assumed that text and illustrations are integrated into one mental model, which tends to be more robust and coherent than mental models generated from text alone (see Cognitive Theory of Multimedia Learning; Mayer, 2021).

To date, only a few studies have investigated the influence of illustrations on updating and knowledge revision processes. Two studies examined how illustrations combined with refutation texts affect the revision of misconceptions (Danielson et al., 2016; Mason et al., 2017). The studies employed different combinations, such as refutation texts with non-refutational illustrations (Danielson et al., 2016) or refutation texts with refutational illustrations (Mason et al., 2017). After reading either the illustrated or the non-illustrated version of the refutation texts, knowledge revision was assessed. In both studies, illustrations did not enhance knowledge revision beyond the effects already elicited by the refutation texts. Since refutation texts typically include a detailed explanation of why the updated information is valid, it is questionable whether illustrations can add further benefit in such cases, as the content of the refutation text itself is likely sufficient to build an elaborate network of the updated information.

A few studies have examined the role of illustrations in debunking misinformation. Across six experiments, Whitehead et al. (2025) found that debunking messages reduced scientific misconceptions, but that illustrations depicting either the misconception or the correct information did not provide any additional benefit. Other findings nevertheless suggest that illustrations can increase the impact of verbal information. Newman et al. (2012) showed that illustrations increased the perceived truth of claims, while Dixon et al. (2015) found that illustrations supported the communication of weight-of-evidence information about the alleged link between vaccines and autism. Taken together, the findings are mixed about the role of illustrations in debunking misinformation.

Previous research on refutation texts and misinformation debunking has primarily assessed the outcomes of updating using measures such as knowledge tests and subjective judgments of truth. These measures indicate whether updating has occurred but provide limited insight into whether and how illustrations influence cognitive processing during reading. Process measures, such as reading and response times, may provide a more direct indication of whether illustrations influence the activation of updated and outdated information during comprehension.

Frick et al. (2025) addressed this issue using reading times. Participants read texts containing outdated information, which was later updated as the narrative progressed. The updated information was presented either with or without an illustration. Additionally, they included control texts that were fully consistent from the beginning, and either illustrated or not. Illustrations generally facilitated target-sentence reading but did not specifically reduce the reading-time disruption caused by outdated information, as the predicted interaction between updating and illustration was not significant. This suggests that illustrations may enhance the activation of the information they illustrate (e.g., the updated information), which in turn facilitates reading the target sentence. At the same time, this stronger activation of the updated information appears not to suppress the activation of the outdated information, at least the interaction did not reach significance. So, while the activation of illustrated updated content is strengthened, the outdated information remains active enough to still influence the processing of the target sentence.

A key limitation of the study by Frick et al. (2025) is that reading times for the target sentence reflect disruptions in reading and comprehension but are less sensitive to the activation process itself (see Kendeou et al., 2013). This may explain why the interaction failed to reach significance, despite the descriptive data trending in the expected direction (see Frick et al., 2025, Experiment 1).

The aim of the present study is, therefore, to examine the effect of illustrations on the activation of the illustrated information in a more targeted manner. To this end, we employed the statement verification task, which had been used by Kendeou et al. (2013).

### The present experiments

The aim of the present experiments was to investigate the effect of illustrating updated information on activation processes, thereby testing the original assumptions by Frick et al. (2025) by using a different paradigm. We assumed that illustrating the updated information would enhance its activation while simultaneously reducing the activation of the outdated information. Accordingly, when participants read a target sentence explicitly referring to the updated information, the outdated information should be less strongly reactivated if the updated information had previously been illustrated than if it had not been illustrated.

In our experiments, we employed the statement verification task used by Kendeou et al. (2013). Verification statements referring to the outdated information were presented either directly before (i.e., after the background section) or directly after the target sentence. Faster responses after than before the target sentence indicate that the target sentence reactivated the outdated information. We predicted that this response-time difference would occur when the updated information was not illustrated but would be reduced or absent when it was illustrated. As the verification statement task is more sensitive to activation processes (Kendeou et al., 2013), it may be better suited than reading times (cf. Frick et al., 2025) to show an interaction related to activation processes.

Both experiments used a 2 × 2 within-subjects design with the factors illustration (updated information illustrated versus not illustrated) and position (verification statement presented before versus after the target sentence). In both experiments, we predicted an interaction between illustration and position. For non-illustrated texts, verification statement responses were expected to be faster after than before the target sentence, indicating reactivation of the outdated information. For illustrated texts, we expected no response-time difference between the two positions, indicating that illustrating the updated information reduced or eliminated the later reactivation of the outdated information. The experiments differed in how the updated information was presented. In Experiment 1, the updated information was presented in a matter-of-fact manner. In Experiment 2, it was supplemented with a one-sentence causal explanation for the change. This modification was motivated by findings showing that causal explanations can strengthen updated information, although Kendeou et al. (2013) found that a single causal sentence did not eliminate the later reactivation of outdated information, whereas a more elaborate three-sentence causal explanation did. We nevertheless reasoned that combining the illustration with a causal explanation might provide converging sources of elaboration and therefore strengthen the updated information sufficiently to reduce activation of the outdated information. Accordingly, the same illustration × position interaction was predicted in both experiments, but Experiment 2 tested whether this effect would emerge when the illustrated update was additionally supported by a causal explanation.

The materials, data, and analyses codes are available under the following link: https://osf.io/8d36c.

## Experiment 1

Experiment 1 tested whether illustrating updated information reduces the later reactivation of outdated information when the update is presented in a matter-of-fact manner. In contrast to Frick et al. (2025), the experiment included only outdated text versions and no consistent text versions. The experiment was pre-registered (see https://aspredicted.org/sjpw-njnk.pdf) and approved by the local ethics committee.

### Method

#### Participants and design

There were four different conditions resulting from crossing the factors position (verification statement after background section vs. verification statement after target sentence) and illustration (non-illustrated vs. illustrated). The conditions were varied within participants.

We conducted a power simulation based on the data of Kendeou et al. (2013) and Frick et al. (2025). Since these two studies did either not include the same measure as the current study (Frick et al., 2025) or did not include the factor illustration (Kendeou et al., 2013), the effect sizes had to be estimated as well as possible. The fixed effects were set as follows: position verification statement = -150 ms, illustration = -40 ms, interaction of position verification statement and illustration = 120 ms. The *SD* of the random effects were set as follows: participant = 600 ms, item (text passage) = 150 ms, residual = 750 ms. The simulations (*N* = 2000) revealed that 320 participants were necessary for sufficient power (1- β > 0.8).

Taking possible exclusion of participants into account, 350 participants were recruited via Prolific. However, the exclusion based on our pre-registered exclusion criteria was higher than expected and additionally 30 participants were recruited after the first data collection. In sum, 380 participants were recruited and received 7.70 pounds for their participation. The screening criteria were English language proficiency (good level or above), age (between 18 and 35 years), handedness (right), current country of residence (USA), and no language related disorder.

In total, 58 participants were excluded. For one person, data-saving was not working. Three persons were excluded based on self-reported technical issues. Two persons reported only average language skills, and three persons were older than 35. Furthermore, 47 participants answered more than 40% of the attention-check items erroneously (see below for details). Finally, two participants were excluded from the analysis as they answered all verification statements extremely fast (< 500 ms), extremely slow (> 8000 ms), or wrong. These borders were pre-registered beforehand. In sum, 322 participants remained for the analyses (154 female, 158 male, 9 non-binary, 1 none of these options; *M* = 28.13 years, *SD* = 4.48).

#### Materials

In total, 16 experimental text passages, already used in previous experiments (Frick et al., 2025), were included in the current experiment. A full-text sample can be seen in Table 1. All text passages required updating and consisted of a short introduction, an elaboration section, a background section, and, depending on the condition, a target sentence. For example, one text passage was about Terry, who wants to buy an instrument. In the music store, Terry first sees an oboe, which she wants to buy (outdated information). However, shortly before buying it, she decides to buy a cello instead (updated information). In the conditions in which the verification statement was presented after the target sentence, a target sentence referring to the updated information was presented (e.g., “Terry showed her the cello she bought.”). In conditions where the verification statement was presented directly after the background section, we did not present the target sentence.

For every text passage, a verification statement, referring to the outdated information, was formulated, e.g., “Terry initially intended to buy an oboe.” These verification statements were controlled for length on character level (*mean* = 39.31 characters, range = 37–40). All verification statements for the experimental stories were true and required a yes answer. It was varied whether this verification statement was presented after the background section or after the target sentence.

In the illustrated conditions, the sentence conveying the updated information was illustrated. To mask the research aim, two additional sentences were also illustrated. All illustrations were drawn by the research team and illustrated the information presented in the sentence. Each illustration was presented above the corresponding sentence (see Table 1).

In sum, each text passage existed in four conditions: non-illustrated text passage with the verification statement after the background section, non-illustrated text passage with the verification statement after the target sentence, illustrated text passage with the verification statement after the background section, and illustrated text passage with the verification statement after the target sentence. To counterbalance the different versions of each text passage, four stimuli lists were generated. Each list contained 16 experimental text passages. Each text passage was presented once within the same list. Within each list, the conditions were evenly distributed (four text passages in each condition). Across all lists, each version of each text passage was presented. Additionally, 16 filler passages were included in these lists. Eight of these filler passages were illustrated, and eight were non-illustrated. All verification statements for the filler passages were wrong and required a no answer. Therefore, yes and no answers were required equally often within each stimuli list. The 32 text passages within each list were presented randomly.

**Table 1:**
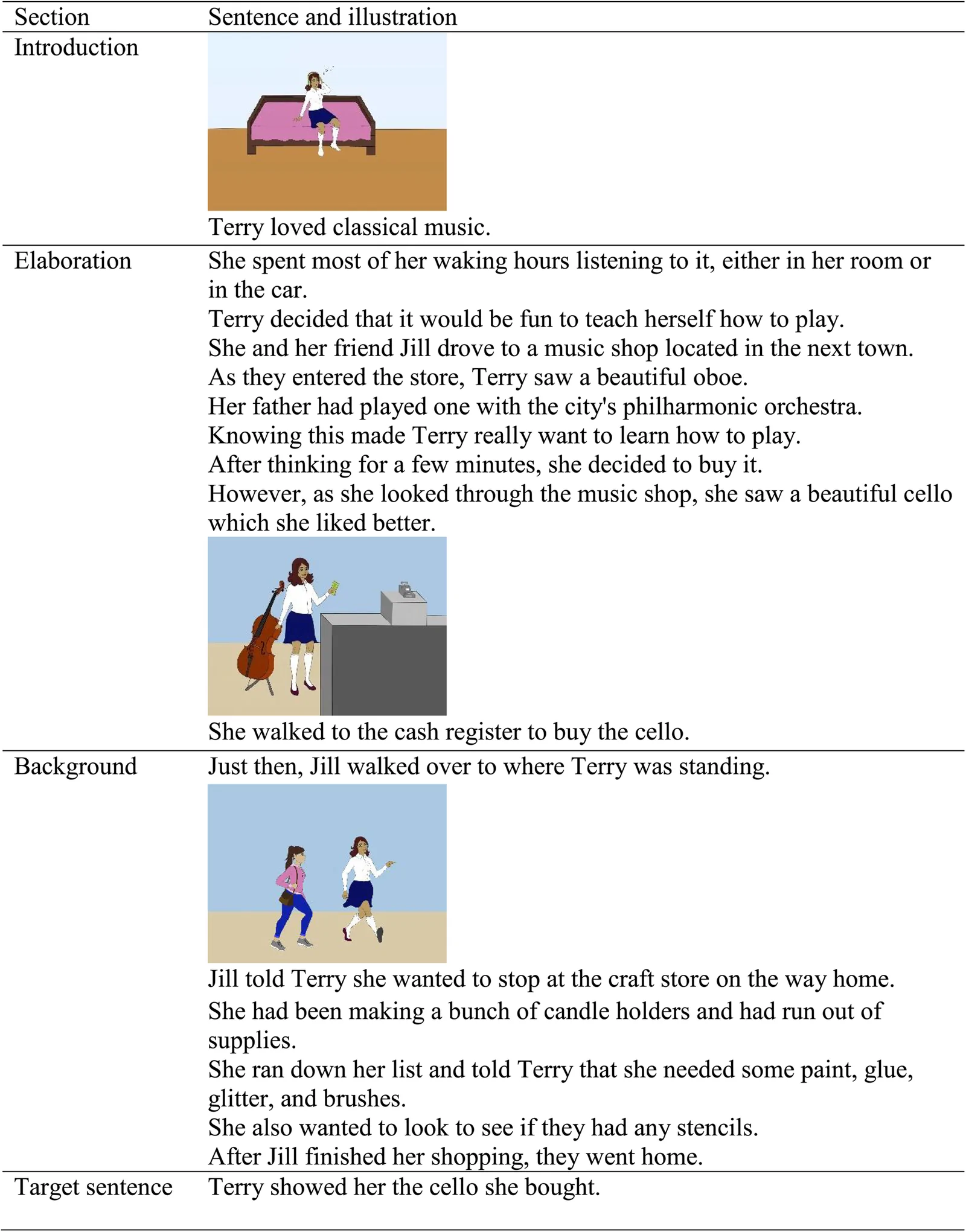
Example Text Including the Illustrations

Note. The text was presented sentence by sentence. The illustrations were only presented in the illustrated conditions. In this case, each illustration was shown above the corresponding sentence (see, for example, the ‘Introduction’ row in the table). The verification statement (“Terry initially intended to buy an oboe”) was either presented after the last sentence of the background section or after the target sentence. If the verification statement was presented after the background section, the target sentence was not presented at all

#### Measures

##### Dependent measures

We measured the response time to answer the verification statements. If the answer to the verification statement was yes, participants should press the k key. If the answer was no, participants should press the d key. Only correct answers (= yes answers) were included in the analysis.

##### Attention-check measures

A text attention-check measure and an illustration attention-check measure were included. For the text attention-check measure, we presented a second verification statement (yes/no answer) after each text passage (*n* = 32). This second verification statement was always presented after the first verification statement. The attention check item referred to the general content of the text passage and never to the outdated/updated information, e.g., “Terry liked classical music.” Again, if the answer to the item was yes, participants should press the k key, if it was no, they should press the d key. Overall text attention check measure, yes and no answers were required equally often.

For the illustration attention check, we included an illustration memory task. After all text passages had been presented, we presented 32 illustrations individually on the screen. Participants had to decide whether they had seen the illustration before or not. Sixteen illustrations were old; participants had seen them before; 16 illustrations were new, and participants had not seen them before. If participants had not seen the illustration before, they should press the d key; if they had seen it before, they should press the k key.

As pre-registered, participants with 13 or more incorrect answers in one or both attention check measures were excluded from the analysis. After excluding these participants, the remaining participants answered an average 29.12 (*SD* = 2.63; 91.00%) of the text attention check items and 26.63 (*SD* = 3.19; 83.22%) of the illustration attention check items correctly.

#### Procedure

The experiment was implemented in PsychoPy/PsychoJS (Peirce et al., 2019) and conducted online. Participants were randomly assigned to one of the four stimuli lists. Before the experiment, participants read general information about the procedure, provided informed consent for study participation and data processing, and answered demographic questions regarding age, gender, English language skills, handedness, and educational level. After giving their informed consent, participants learned that they were about to read several short stories, some of them illustrated, some of them non-illustrated. Further, they learned that the stories were presented to them sentence by sentence, and whenever they pressed the space bar, the next sentence (and if applicable), the next illustration would appear on the screen. After each short story, they should decide on two statements, whether they were true or false, regarding the story they just read. The first statement was always the experimental verification statement, and the second statement was the text attention check item. The instruction emphasized that it was important to answer these statements as fast and accurately as possible. After that, participants learned that they should place their right thumb on the space bar, the right index finger on the k key, and the left index finger on the d key. Before the experimental passages were presented, participants read two practice stories (one illustrated, one non-illustrated) to get used to the procedure. After the practice round, participants were reminded to place their fingers as instructed and to read at a normal reading speed.

Each experimental passage started with the words “This is story number N. Please press the space bar to start it” (*N* = number of the story). Then, with every press of the space bar, the current sentence (and illustration, if present) disappeared and the next sentence appeared. After the last sentence of each story (depending on the condition, either after the background section or after the target sentence), a fixation cross appeared in the middle of the screen for 500 ms. Then, the verification statement was presented. After answering that item, participants received feedback on whether they answered the item correctly or incorrectly: the word “correct” or “wrong” was written in the middle of the screen for 1500 ms. After that, a new fixation cross was presented for 500 ms in the middle of the screen, before the text attention check item was presented. Again, participants received feedback on the accuracy of their answer.

After all 32 text passages (experimental and filler passages), participants received the instructions for the illustration memory task. The procedure was the same as before: participants pressed the k key for yes and the d key for no answer. No feedback was provided for the pictorial attention-check task. After completing this task, participants answered questions about possible technical problems, were given the possibility to write a comment about the experiment, were debriefed, thanked, and asked to confirm their consent for data processing. The whole procedure lasted about 45 min.

### Results

The data for this and the following experiment were analyzed with R, version 4.3.2 (R Core Team, 2023). The linear mixed effects model was fitted to the data using the lme4 package (Bates et al., 2015); significance values were computed with the lmerTest package (Kuznetsova et al., 2017).

Following our pre-registration, we excluded responses with exceptionally fast (< 500 ms) or slow response times (> 8000 ms.). This resulted in the exclusion of 148 trials. Furthermore, only correct answers were included, which resulted in the exclusion of 746 trials (approx. 14%). The final sample size was 4258 trials.

Table 2 lists the mean response times to the verification statements as a function of the factors position and illustration.

**Table 2:** Mean Response Time (in s) and Standard Deviations (in Brackets) to the Verification Statement as a Function of the Factors Verification Statement Position and Illustration in Experiment 1

Position	Illustration	
	Non-illustrated	Illustrated
After background section	2.35 (1.14)	2.36 (1.13)
After target sentence	2.26 (1.14)	2.31 (1.18)

Since the data had a heavy-tailed distribution, we log-transformed the response times^1^. We then fitted a linear mixed effect model with participants and text passages (item) as random effects (random intercept) and position (after the background section = 0, after the target sentence = 1) and illustration (non-illustrated = 0, illustrated = 1) as fixed effects to the data. Table 3 lists estimates and significance values of the fixed effects. The simple effect of the factor position was significant (see Table 3 for statistical values). Response times were significantly faster when the verification statement was presented after the target sentence (*M* = 2.29, *SD* = 1.16) than when it was presented after the background section (*M* = 2.35, *SD* = 1.13). Contrary to our hypothesis, the illustration did not influence the response time. There was neither a significant simple effect of the factor illustration nor a significant interaction effect between the factors position and illustration.

**Table 3:** Fixed and Random Effects of the Linear Mixed Effects Model for the Response Times

	Estimate	SE	df	t	*p*
Intercept	0.78	0.02	214.6	329.39	< 0.001
Position	-0.04	0.01	3921	-3.00	0.003
Illustration	0.01	0.01	3920	1.21	0.228
Position * Illustration	-0.01	0.02	3920	-0.42	0.675
**Random Effects**					
*Group*	*Effect*	*Variance*	*SD*		
Participant	Intercept	0.12	0.35		
Item	Intercept	< 0.01	0.04		
Residual	-	0.08	0.28		

### Discussion experiment 1

In sum, we were able to replicate the findings reported by Kendeou et al. (2013): Participants were faster in verifying the statement about the outdated information after reading the target sentence than after reading the background sentence. Contrary to our expectations, this pattern was not influenced by the illustration. Response times were faster after the target sentence than after the background sentence, independently of whether the updated information was additionally illustrated with an illustration. Hence, it seems that illustrating the updated information with an illustration was not sufficient to eliminate the activation of outdated information during reading the target sentence.

A critical review of the materials used in Experiment 1 revealed two aspects that could be improved. First, the updated information was presented in a matter-of-fact manner, merely stating that the protagonist chose the updated rather than the outdated option without specifying the reason for this change (e.g., Terry decided to purchase a cello instead of an oboe). Second, the verification statements were not fully consistent in terms of sentence construction, length, and the position of the critical word.

We addressed both aspects in Experiment 2. The updated information was supplemented with a one-sentence causal explanation specifying the reason for the change from the outdated to the updated information. In addition, the verification statements were revised to increase their consistency: The critical word was always placed in the final position, four sentence constructions were used with equal frequency, and statement length was controlled at the character level. Experiment 2 thus examined the effect of illustrating causally explained updated information using more consistent verification statements.

## Experiment 2

Experiment 1 did not provide evidence that illustrating the updated information reduced the reactivation of outdated information during target-sentence processing. Experiment 2, therefore, examined whether the predicted effect would emerge when the updated information was additionally supported by a one-sentence causal explanation. We reasoned that illustrations and the causal explanation might jointly provide a more elaborated representation of the updated information. The design and predicted illustration × position interaction were otherwise identical to those of Experiment 1. The experiment was pre-registered (https://aspredicted.org/k92q-bvmy.pdf) and approved by the local ethics committee.

### Methods

#### Participants and design

We used the same within-subjects study design as in Experiment 1: illustration (non-illustrated vs. illustrated) and position (verification statement after the background section vs. verification statement after the target sentence). This resulted in four different experimental conditions varied within a participant.

We conducted a power simulation with parameters similar to those used in Experiment 1 but adjusted according to empirically observed patterns. To re-evaluate the relevance of the initial simulation set-up, we built the same mixed-effects model described in Experiment 1, this time using raw (instead of log-transformed) data as input. A further comparison between the empirically observed values and the values used in the power simulation showed that the largest discrepancy between the expected and empirical results (-40 ms and 30 ms, respectively) occurred for the illustration effect—that is, illustration presentation led to descriptively slower response times. Similar to the findings of Kendeou et al. (2013, Experiment 5), where a three-sentence causal explanation increased response times by 95 ms compared to a one-sentence explanation (averaged across positions), we expected even more prolonged response times in Experiment 2 due to the combined influence of illustrations and causal explanations. Accordingly, we adopted 95 ms as the new predicted value for the illustration effect, in line with Kendeou et al.’s findings. Position effect was estimated as the mean of expected (-150) and observed (-71) values in Experiment 1. Interaction of illustration and position was set up at 110, since, as mentioned before, we expected no difference in the response time as a function of the factor position for illustrated texts. These adjustments resulted in the following fixed effects set-up: illustration = 95 ms, position verification statement = -110 ms, and interaction of illustration and position verification statement = 110 ms.

Standard deviations of random effects were based on Experiment 1 results, with slight reductions expected due to increased homogeneity of the verification statements (see below): participant = 900 ms, item (text passage) = 70 ms, and residual = 750 ms. According to simulation results (*N* = 2000), 365 participants were necessary to achieve sufficient study power (1- β > 0.8). To account for the potential exclusion of participants, we aimed to collect data from 400 persons. However, after data preprocessing, the sample was reduced to 321 participants. Thus, data from 48 additional participants were collected.

Participants were recruited via Prolific and received 6.84 pounds for their participation. The screening criteria were no participation in Experiment 1, Prolific approval rate of 100%, English language proficiency (good level or above), age (18–35 years), handedness (right-handed), current country of residence (USA), and no language-related disorders.

After data preprocessing, 94 participants were excluded. For four participants, data-saving was not working. One person did not agree to process their data at the end of the study. One person reported severe technical issues occurring over the course of the experiment. Two persons had only average language skills. One person did not report their age, and three were older than 35 years. Moreover, 79 participants did not pass one or both of the attention checks. Finally, three participants were excluded from the analysis as they answered all verification statements extremely fast (< 500 ms), extremely slow (> 8000 ms), or wrong. As a result, the final sample included 354 participants (147 female, 201 male, 6 non-binary; *M* = 27.36 years, *SD* = 5.04). Although the final sample size was slightly lower than preregistered (354 vs. 365 participants) due to organizational constraints, an a posteriori sensitivity analysis indicated that the achieved sample still provided adequate statistical power (1-*β* = 0.797), supporting the robustness of the reported findings.

#### Materials

The materials consisted of the 16 experimental text passages used in Experiment 1 that were slightly modified according to the aim of the current study. The updated information was causally explained. The one-sentence causal explanations followed a consistent structure across stories, specifying the reason for the change from the outdated to the updated information. For example, in Terry’s story, the following explanation was provided: “However, she remembered that her father had always dreamed of them playing together as a band”. After introducing the causal explanation, the protagonist’s action with the preferred, updated object was described in the second sentence. For example, in Terry’s story, the following information was provided: “So, she decided to buy a cello instead to accompany him.” This part of the passage was followed by the background section unrelated to the updated information (see Table 1). Finally, depending on the experimental condition, a target sentence referring to the updated information was presented.

Besides adding causal explanations, minor adjustments were made to the stimuli. Specifically, we changed the outdated objects mentioned in three stories. In one story, this change was crucial for a causal explanation. In two other stories, we replaced objects with single-word names (e.g., “a leafy plant” was replaced with “a violet”). Finally, a few grammar and content-related adjustments were made where needed to improve the clarity of the causal version of the story. After these adjustments, the text passages ranged in length from 162 to 233 words (*M* = 200.56, *SD* = 24.65 words).

Depending on the experimental condition, participants were presented with the verification statements (e.g., “*Terry initially intended to buy an oboe.*”) containing the outdated information after the background section or after the target sentence. We slightly modified the verification statements used in Experiment 1 to ensure higher consistency. After modification, the critical word was always placed in the final position of the verification statement and four different sentence constructions (“*At first*,* someone did something with [critical word]*”, *“Someone at first did something [critical word]”*, *“Initially*,* someone did something [critical word]”*, and “*Someone initially did something [critical word]*”) were used with equal frequency. The length of verification statements was controlled at the character level (37–40 characters), with an average length of *M* = 38.25 (*SD* = 1.06) characters. All the verification statements were true and counterbalanced by false verification statements for 16 filler stories.

The same illustrations as in Experiment 1 were used. It was again varied whether the text passages were presented with or without illustrations. Participants were again randomly assigned to one of four counterbalanced stimuli lists. For each list, all items were randomized during the presentation.

#### Measures

##### Dependent variable

The dependent variable was the same as in Experiment 1.

##### Attention-check measures

The attention-check measures were the same as in Experiment 1. After excluding participants exceeding 13 or more wrong answers in one or both attention-check measure, the remaining participants answered an average 28.93 (SD = 2.26; 90.40%) of the text attention check items and 26.42 (SD = 3.20; 82.56%) of the illustration attention check items correctly.

#### Procedure

The procedure was the same as in Experiment 1.

### Results

Again, we excluded trials with extremely fast (< 500 ms) and extremely slow (> 8000 ms) responses, which led to the exclusion of 279 trials. For the main analysis, we selected only trials with correct answers (i.e., yes), resulting in the removal of 921 trials (approx. 16%). Thus, the final dataset consisted of 4464 trials.

The average time for responding to the verification statements under different experimental conditions is presented in Table 4.

**Table 4:** Mean Response Time (in s) and Standard Deviations (in Brackets) to the Verification Statement as a Function of the Factors Verification Statement Position and Illustration in Experiment 2

Position	Illustration	
	Non-illustrated	Illustrated
After background section	2.70 (1.38)	2.68 (1.34)
After target sentence	2.60 (1.33)	2.63 (1.35)

Since the data had a heavy-tailed distribution typical of reaction time measures, it was, as pre-registered, log-transformed before analysis. The same linear mixed effects model as in Experiment 1 was used.

Table 5 summarizes the estimated model parameters. The results replicated the effects observed in Experiment 1. There was a significant simple effect of the position of the verification statement: participants responded faster when the item was presented after the target sentence (*M* = 2.61, *SD* = 1.34) compared to when it was presented after the background section (*M* = 2.69, *SD* = 1.36). Unexpectedly, illustrations did not influence participants’ performance. Neither the simple effect of the illustration nor its interaction with the position of the verification statement was significant.

**Table 5:** Fixed and Random Effects of the Linear Mixed Effects Model for the Response Times

**Fixed Effects**					
*Effect*	*Estimate*	*SE*	*df*	*t*	*p*
Intercept	0.90	0.03	117	299.96	< 0.001
Position	-0.03	0.01	4099	-2.40	0.016
Illustration	0.01	0.01	4099	1.03	0.302
Position * Illustration	-0.01	0.02	4098	-0.35	0.724
**Random Effects**					
*Group*	*Effect*	*Variance*	*SD*		
Participant	Intercept	0.14	0.37		
Item	Intercept	< 0.01	0.06		
Residual	-	0.09	0.30		

### Discussion experiment 2

The results of Experiment 2 replicated the effects observed in Experiment 1. Specifically, participants responded faster to verification statements when they were preceded by the target sentence. This suggests that the causal explanations provided to participants in Experiment 2 were not sufficient to eliminate the activation of outdated knowledge, as the simple effect position was still observed. Moreover, contrary to our hypothesis, adding illustrations to the experimental stories did not influence participants’ performance. Thus, unlike the additional three-sentence causal information in Kendeou et al. (2013, Experiment 5), the illustrations added to the one-sentence causal information did not help to suppress the activation of outdated information.

Compared to Experiment 1, response times in Experiment 2 were, on average, approximately 300 ms longer. One possible explanation is that the updated information in Experiment 2 was more elaborated due to the additional causal explanations, which may have made it more difficult to retrieve the outdated information, resulting in longer response times.

Another difference between the experiments lay in the construction of the verification items. In Experiment 2, the critical word referring to the outdated information consistently appeared at the end of the item. This positioning may have led to longer response times, as the entire item had to be processed in order to make a decision about its correctness.

However, since these factors were not experimentally manipulated in a single experiment, this interpretation remains speculative.

## General discussion

Research has shown that outdated information can be automatically activated even after being updated by current, valid information, leading to disruptions in the reading process and hindering comprehension (Johnson & Seifert, 1994; Lewandowsky et al., 2012; O’Brien et al., 1998, 2010; Rich & Zaragoza, 2016). The goal of the reported experiments was to investigate whether illustrating the updated information could strengthen its activation and effectively suppress the influence of outdated information. To answer this question, we used the verification statement task introduced by Kendeou et al. (2013). In this task, verification statements referring to the outdated information (e.g., “Terry initially wanted to buy an oboe”) are presented either immediately before or after the target sentence (e.g., “Terry showed her the cello she bought”). If the outdated information is automatically activated while reading the target sentence, participants should respond to the verification statement more quickly after the target sentence than before it, as the relevant information would already be active in memory. We therefore expected this response-time difference to occur when the updated information was not illustrated, but to be reduced or absent when it was illustrated.

To keep it brief: We were unable to confirm this hypothesis in either Experiment 1 or Experiment 2. With or without an illustration, verification statements were answered more quickly when presented after the target sentence in both conditions. Thus, illustrations did not suppress the activation of the outdated information while reading the target sentence, in either experiment. This finding is consistent with the results reported by Frick et al. (2025), who observed faster reading times for the target sentence when updated information was illustrated, but were likewise unable to eliminate the influence of outdated information. That is, outdated information continued to be activated during the reading of the target sentence.

Since we did not measure reading times for the target sentence, we cannot determine whether it was processed more quickly in the illustrated condition compared to the non-illustrated condition. However, our data suggests that outdated information was likely retrieved during the reading of the target sentence, even in the illustrated condition. Neither the illustration of the updated information alone (Experiment 1) nor its combination with a one-sentence causal explanation(Experiment 2) was sufficient to eliminate the retrieval of outdated information. This suggests that illustrations, at least as implemented in the reported experiments, may not be effective in strengthening the updated information to a degree that prevents the activation of outdated content.

What might explain this? One possible reason is that the illustrations were not adequately processed by participants. Indeed, eye-tracking studies have shown that readers tend to focus primarily on the text and often overlook accompanying illustrations (Jian & Wu, 2015; Johnson & Mayer, 2012; Schmidt-Weigand et al., 2010). If this was the case, the illustrations would not have been able to exert their intended beneficial effects. Since we did not collect eye-tracking data in the present experiments, we cannot directly test this assumption. However, the high exclusion rate of participants who failed the pictorial attention check lends support to this interpretation. Future studies could examine whether strategies such as signaling correspondences between text and illustrations (Richter et al., 2016) or providing self-explanation prompts that encourage participants to process the text and illustration more actively (Berthold et al., 2009) facilitate illustration processing, thereby potentially strengthening updating effects.

Assuming that the illustrations were sufficiently processed by those participants who passed the pictorial attention check (and were included in the analyses), it is still possible that illustrations do not function in the way theoretically assumed. While there is evidence that text and illustration can be integrated into a single mental model (e.g., Arndt et al., 2015; Schüler et al., 2015), it remains unclear whether such integrated mental models strengthen the updated information to a degree that completely suppresses the activation of outdated information. Based on the findings reported by Frick et al. (2025), it appears more likely that illustrated information is indeed reinforced, but not necessarily to the extent that outdated information is no longer retrieved at all.

Moreover, illustrations may not merely affect the activation strength of the updated information, as assumed in our original hypothesis, but may also introduce additional, automatic cross-modal validation processes between the concurrently presented updated textual information and the illustration (Schüler & Frick, 2024) as well as between subsequently presented textual information (i.e., verification statements, target sentence), and the illustrations (Schüler & Frick, 2025). It is conceivable that the additional cross-modal validation processes influenced response times in the illustrated condition, thereby masking a potential facilitative effect of strengthened updated information. Consequently, the absence of an illustration effect on response times does not necessarily imply that the illustration failed to strengthen the updated information. However, because the present paradigm does not allow activation strength and validation processes to be disentangled, this interpretation remains speculative.

Achieving a stronger shift in activation toward the updated information may require combining illustrations with additional textual support. In Experiment 2, the illustration was combined with only a one-sentence causal explanation. This decision was based on the assumption that pictorial and causal information might provide converging sources of elaboration, such that their combination would strengthen the updated representation more than either form of support alone. However, the results provide no evidence for such a cumulative effect. Importantly, Kendeou et al. (2013) found that a single causal sentence was insufficient to eliminate the activation of outdated information; this effect emerged only when the updated information was supported by three causal sentences. Consequently, the null effect in our Experiment 2 may reflect the limited elaboration provided by the one-sentence explanation rather than the general ineffectiveness of combining illustrations with causal explanations. The present findings, therefore, do not allow us to determine whether illustrations could help suppress outdated information when the textual explanation is sufficiently elaborate. Future research should directly compare one- and three-sentence causal explanations, both with and without illustrations, to distinguish the independent and potentially cumulative effects of pictorial and causal elaboration. Studies that combined refutation texts with illustrations likewise failed to observe any positive effects of the illustrations (Danielson et al., 2016; Mason et al., 2017). Research on illustrating debunking messages has also yielded mixed findings regarding whether illustrations enhance the effectiveness of textual corrections (Dixon et al., 2015; Newman et al., 2012; Whitehead et al., 2025). However, this does not preclude the possibility that other forms of textual support, such as repeating the outdated information as part of the correction (Ecker et al., 2017), providing warnings about the outdated information (Ecker et al., 2010), or associating the updated information with a highly credible source (Guillory & Geraci, 2013) in combination with illustrations, could yield stronger effects.

Apart from the missing influence of illustrations, it is important to emphasize that the findings support the theoretical assumptions of both the RI-Val model (O’Brien and Cook 2016a) and the KReC model (Kendeou, 2024; Kendeou & O’Brien, 2014). Consistent with these frameworks, the results indicate that outdated information can be activated even after it has been replaced by updated information. Specifically, when participants read the target sentence, which referred exclusively to the updated information and therefore did not require activation of the outdated information, the outdated information was nevertheless retrieved. This retrieval led to faster response times on the subsequent verification statement compared to when the verification statement was presented before the target sentence. Thus, our study aligns with previous research (e.g., Frick et al., 2025; Guéraud et al., 2005; Kendeou et al., 2013; O’Brien et al., 1998, 2010) demonstrating that outdated information cannot simply be suppressed.

### Limitations

While online studies offer access to a diverse and readily available participant pool, they also come with certain limitations—particularly when conducted via platforms such as Prolific, as in our experiments. One key limitation is the lack of experimental control. Factors such as varying levels of environmental distraction, differences in device usage, and screen size variability can introduce noise into the data, potentially affecting the reliability of the results.

When interpreting the results, it is also important to consider that the experiments used relatively simple texts and illustrations. The effect of illustrations on the updating process may vary depending on the complexity of the material. It is conceivable, for instance, that illustrations exert a stronger influence on updating when the information to be updated is more complex. Many studies comparing illustrated and non-illustrated texts have focused on complex topics, such as the working of brakes or the development of tornadoes (Johnson & Mayer, 2012; Schüler, 2019). It seems plausible that the possible strengthening effects of illustrations are particularly pronounced when the depicted updated information is difficult to describe or explain in text alone.

Experiment 2 was further limited by the use of only a one-sentence causal explanation. Because Kendeou et al. (2013) observed the elimination of outdated information activation only with three causal sentences, our findings cannot establish whether illustrations might be effective when combined with more extensive causal elaboration.

Finally, the simple effect of verification-statement position observed in the present experiments cannot be attributed solely to the activation of outdated information, but may also reflect alternative mechanisms. Because the target sentence contained the updated information, which was conceptually related to the outdated information (e.g., *cello–oboe*), the presence of this conceptually linked content may have been sufficient to strengthen activation of the related concept, independent of its updated or outdated status (i.e., priming). In contrast, the background section did not contain this critical information and therefore could not activate the corresponding conceptual network. However, the findings of Kendeou et al. (2013) argue against this alternative account. In their study, the position effect was eliminated - an outcome unlikely if conceptual co-activation were the primary mechanism. Consequently, given that our study employed the same paradigm, the present results most plausibly reflect the intended activation processes.

To conclude, practitioners should be aware that refuting (mis)information by providing updated information does not necessarily lead to the suppression of the refuted content. Based on the experiments reported here, we conclude that even additional representations, such as illustrations, are unlikely to fully suppress the activation of outdated or retracted information. This finding is consistent with previous studies that have examined the influence of illustrations on strengthening updated information (see, e.g., Danielson et al., 2016; Frick et al., 2025; Mason et al., 2017). Consequently, the recommendation to use illustrations to correct misconceptions (e.g., Brashier & Marsh, 2020) is not yet empirically consistently supported. Therefore, further targeted measures are needed to ensure that misinformation does not continue to influence the processing of new information.

## Data Availability

The materials, data, and analyses codes are available under the following link: https://osf.io/8d36c.

## Footnotes

1 Please note that this transformation was not pre-registered; however, the pattern of results remains consistent with and without the log-transformation.

## Declarations

### Generative AI and AI-assisted technologies in the writing process

While preparing this manuscript, the authors used Grammarly and Copilot to improve language and readability. After using these tools/services, the author(s) reviewed and edited the content as needed and take(s) full responsibility for the content of the published article.
